# Validation of LC-MS/MS Coupled with a Chiral Column for the Determination of 3- or 15-Acetyl Deoxynivalenol Mycotoxins from *Fusarium graminearum* in Wheat

**DOI:** 10.3390/toxins13090659

**Published:** 2021-09-16

**Authors:** Lan Wang, Zheng Yan, Haiyan Zhou, Yingying Fan, Cheng Wang, Jingbo Zhang, Yucai Liao, Aibo Wu

**Affiliations:** 1 SIBS-UGENT-SJTU Joint Laboratory of Mycotoxin Research, CAS Key Laboratory of Nutrition, Metabolism and Food Safety, Shanghai Institute of Nutrition and Health, University of Chinese Academy of Sciences, Chinese Academy of Sciences, Shanghai 200030, China; wanglan@sibs.ac.cn (L.W.); zyan@sibs.ac.cn (Z.Y.); zhouhaiyan2018@sibs.ac.cn (H.Z.); 2 Key Laboratory of Agro-Products Quality and Safety of Xinjiang, Laboratory of Quality and Safety Risk Assessment for Agro-Products (Urumqi), Ministry of Agriculture and Rural Affiairs, Urumqi 830091, China; fyyxass@sina.com (Y.F.); wangchengxj321@sina.com (C.W.); 3 Institute of Quality Standards & Testing Technology for Agro-Products, Xinjiang Academy of Agricultural Sciences, Urumqi 830091, China; 4 College of Plant Science and Technology, Huazhong Agricultural University, Wuhan 430070, China; jingbozhang@mail.hzau.edu.cn (J.Z.); yucailiao@mail.hzau.edu.cn (Y.L.)

**Keywords:** chromatographic separation, chemotype determination, mycotoxin, wheat

## Abstract

The major causal agents *Fusarium graminearum* (*F. graminearum*) and *Fusarium asiaticum* could produce multiple mycotoxins in infected wheat, which threatens the health of humans and animals. Specifically, deoxynivalenol (DON) and its derivatives 3- and 15-acetyldeoxynivalenol (3-ADON and 15-ADON) are commonly detected mycotoxins in cereal grains. However, the good chromatographic separation of 3-ADON and 15-ADON remains challenging. Here, an LC-MS/MS method for the chemotype determination of Fusarium strains was developed and validated. 3- and 15-ADON could be separated chromatographically in this study with sufficiently low limits of detection (LODs; 4 μg/kg) and limits of quantification (LOQs; 8 μg/kg). The satisfying intraday and interday reproducibility (both %RSD_r_ and %RSD_R_ were <20%) of this method indicated good stability. The recoveries of all analytes were in the range of 80–120%. In addition, three *F. graminearum* complex (FGC) strains, i.e., PH-1 (chemotype 15-ADON), F-1 (chemotype 3-ADON) and 5035 (chemotype 15-ADON), were selected to verify the accuracy of the method in differentiating phenotypes. The validation results showed that this LC-MS/MS method based on sample pretreatment is effective and suitable for the chromatographic separation of 3-ADON and 15-ADON in wheat.

## 1. Introduction

**Figure 1:**
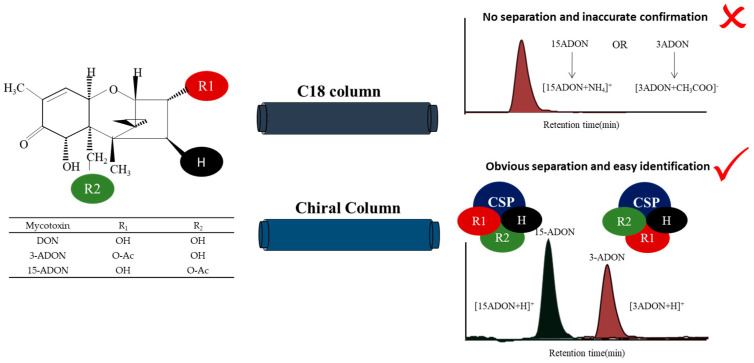
Principle required for the chiral separation of deoxynivalenol (DON), 3-acetyldeoxynivalenol (3-ADON) and 15-acetyldeoxynivalenol (15-ADON).

Wheat is one of the three major food crops around the world, in addition to rice and maize. Trichothecene mycotoxins, especially deoxynivalenol (DON; Figure 1), infect wheat crops, causing Fusarium head blight (FHB) [1,2]. FHB is one of the most destructive global diseases and is responsible for significant wheat crop losses [3]. In China, FHB epidemics occur frequently along the middle and lower reaches of the Yangtze River, especially in Hubei, Anhui and Jiangsu Provinces, resulting in huge yield losses for farmers every year [4,5]. In addition to yielding losses, fungal mycotoxins, which have high toxicity in humans and animals, are produced during the growth process [6,7]. Studies have shown that the *Fusarium graminearum* (*F. graminearum*) complex (FGC), the main pathogen of FBH, includes *F.* *graminearum* and *Fusarium asiaticum* (*F. asiaticum*) [4,8]. The FGC can also be divided into different chemical types (nivalenol (NIV), 3-acetyldeoxynivalenol (3-ADON; Figure 1), 15-acetyldeoxynivalenol (15-ADON; Figure 1), etc.) according to the different types of toxicants produced [9,10,11].

DON may contribute to risk of acute human symptoms, including nausea, gastrointestinal upset, vomiting and diarrhoea [12]. Human could be exposed to DON easily, and dietary intake is thought to be a key aspect in DON exposure. Acetylated DONs show a stronger toxicity than DON, because they are absorbed more rapidly into the intestine [13]. A previous study indicated that permeability and IL-8 secretion are highest in human intestinal cells when exposed to 15-ADON [14]. There is increasing evidence that the potential risk to animals and human in the presence of DON and DON derivative could be individual or additive. Therefore, their contamination is considered as a growing public concern. Taken together, the previous provisional maximum tolerable daily intake (PMTDI) of 1 μg/kg bw/day for DON was changed to a group PMTDI for the three compounds by the Joint FAO/WHO Expert Committee on Food Additives (JECFA) in 2010 [15].

The most common chromatographic methods for the determination of type B trichothecenes include gas chromatography (GC), gas chromatography with mass spectrometry (GC/MS), high-performance liquid chromatography (HPLC) and LC-MS/MS [16,17,18,19]. Recently, LC-MS/MS has been considered the most widely used analysis technique to determine multiple mycotoxins in wheat and wheat-based products [20,21,22]. In particular, LC-MS/MS methods have been developed to evaluate the risk of DONs from wheat and cereals to human health [23,24,25]. However, they are not able to achieve the full chromatographic separation of 3-ADON and 15-ADON, and thus, the reported values indicate the sum of 3-ADON and 15-ADON. Nonetheless, because of their importance, the discrimination of 3-ADON and 15-ADON continues to attract considerable attention.

To minimize the number of treatment samples while preventing exposure to matrix effects, a quick, easy, cheap, effective, rugged and safe (QuEChERS) method is a suitable approach. This simple and effective method is applied to extract mycotoxins from different foods in both solid and liquid samples [26,27].

In mass spectrometry, the discrimination of 3-ADON and 15-ADON is a challenging endeavour, since enantiomeric isomers generally have identical mass-to-charge ratios (*m*/*z*), rendering them indistinguishable. In the research by Girolamo et al., three laboratories wer not able to resolve 3- and 15-ADON peaks, and only the sum of the two isomers was obtained [23]. Isotopically labelled internal standards were used in their study to achieve a satisfactory precision. However, only 3-ADON was detected with the response ratio of the isotopically labelled samples, since 3-ADON and 15-ADON were detected in negative and positive ion modes, respectively, in two separate chromatographic runs. The data from their study confirmed that there is a recognized need for the good chromatographic separation of 3-ADON and 15-ADON. For example, in a previous study, it was observed that 15-ADON formed an [M + NH_4_]^+^ adduct in positive electrospray ionization (ESI^+^) mode, which had a higher abundance than the [M + CH_3_COO]^−^ adduct observed in ESI^−^ mode [28]. However, the positional isomers 3-ADON and 15-ADON could not be separated chromatographically due to their low separation efficiency on the HPLC column. We all know that stable complexes are required to form by the host and guest reasonably in the process of chiral discrimination. The chiral stationary phase (CSP) method is based on the energy difference or stability difference between the analytes and the chiral selector on the surface of the stationary phase, forming temporary noncorresponding isomer complexes. This method allows the direct resolution of diastereomers without transformation. The “three-point attachment” model (Figure 1) combines these interactions and has long been employed in the condensed phase to understand enantioselectivity, which more generally describes the nature of the interactions [29]. Therefore, CSPs were selected to separate chiral drugs and intermediates in recent years [30,31,32].

The ability to produce mycotoxins under certain conditions has been reported to be a strain-specific characteristic. Indeed, research in this area has explored the genetic background of *F. graminearum*, *F. asiaticum* and related species [33,34,35], but genetic variation among isolates belonging to the FGC is extensively described. Variations in the culture characteristics and the aggressiveness and type of mycotoxins have been identified among samples isolated in the same field [36,37]. Determining mycotoxin production and chemotype is fundamental to understanding the contamination level of the strain. Therefore, the development of a valid method to determine the mycotoxin production and chemotypes of fungal strains is significant for providing new insights into the development of comprehensive and effective disease and mycotoxin management strategies.

The objective of this study was to develop a method for the chromatographic separation of 15-ADON and 3-ADON by LC-MS/MS in wheat. In our study, the isomers 3-ADON and 15-ADON could achieve the full chromatographic separation in a chiral column with lower limits of detection (LODs) and limits of quantification (LOQs). Furthermore, the mycotoxin production of strains 5035, PH-1 and F-1 was validated using the candidate method, which has the merits of high throughput, desirable sensitivity, and recovery. This quick, easy and effective assay will contribute to the 3-ADON and 15-ADON determination and mycotoxin management and will promote exposure assessment.

## 2. Results and Discussion

### 2.1. Optimization of LC Conditions

**Figure 2:**
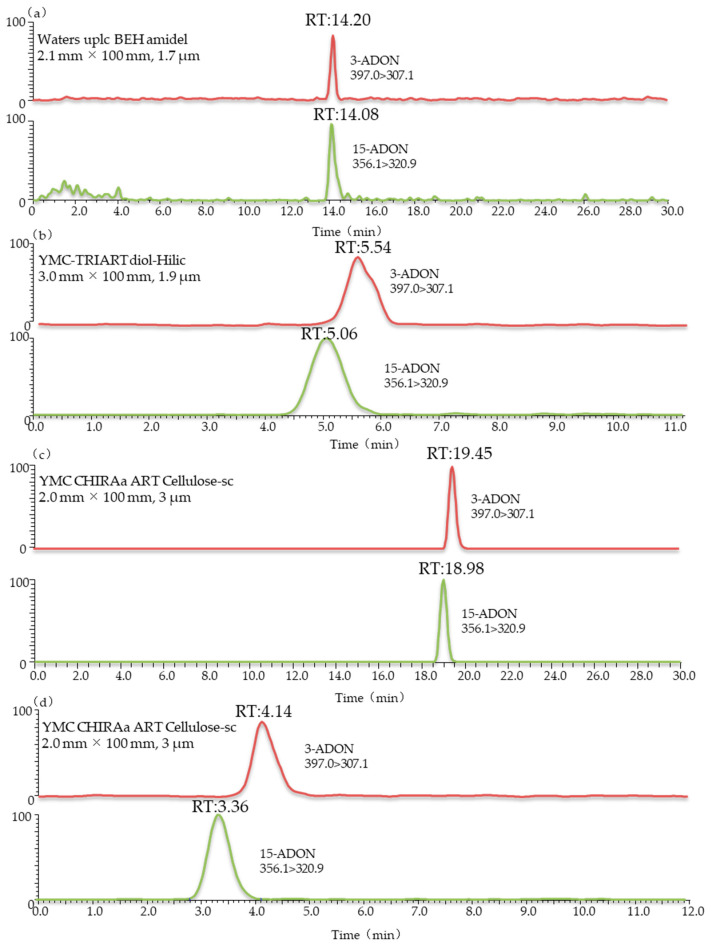
LC-MS/MS chromatograms of wheat blank samples spiked with 3-ADON and 15-ADON using different columns: (**a**) waters UPLC BEH amidel (2.1 mm × 100 mm, 1.7 μm); (**b**) YMC-TRIART diol-HILIC (3.0 mm × 100 mm, 1.9 μm); (**c**) YMC CHIRAa ART Cellulose-sc (2.0 mm × 100 mm, 3 μm); (**d**) YMC CHIRAa ART Cellulose-sc (2.0 mm × 100 mm, 3 μm). Eluent A: 5 mM ammonium acetate; eluent B: methanol.

**Figure 3:**
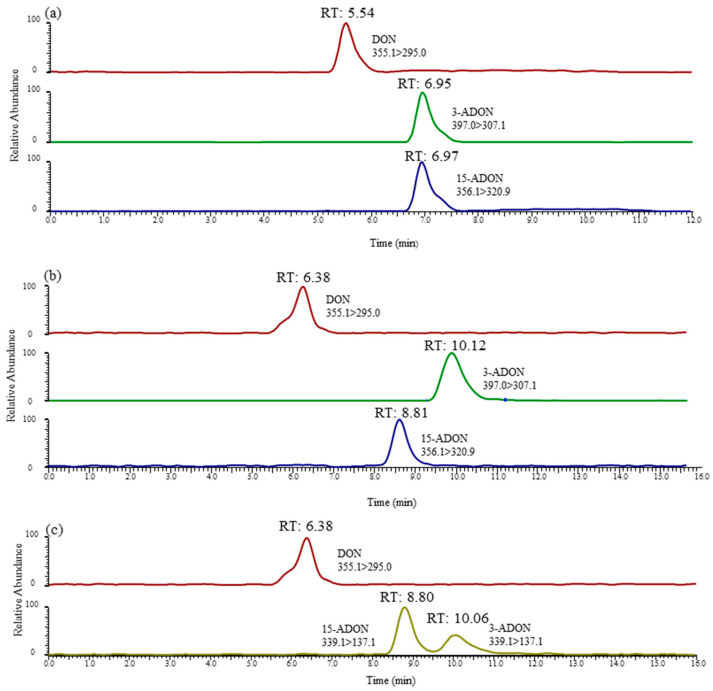
LC-MS/MS chromatograms of DON, 3-ADON and 15-ADON in method A (**a**), method B (**b**) and method C (**c**). DON and 3-ADON had a concentration of 8 ng/mL, and 15-ADON had a concentration of 16 ng/mL.

The Agilent Extend-C18 (3.0 mm × 150 mm, 3.5 μm), Waters UPLC BEH amidel (2.1 mm × 100 mm, 1.7 μm), YMC-TRIART diol-HILIC (3.0 mm × 100 mm, 1.9 μm), YMC CHIRAa ART Cellulose-sc (2.0 mm × 100 mm, 3 μm) and YMC CHIRAa ART Cellulose-sc (3.0 mm × 250 mm, 3 µm) columns were used for separation in this study. Comparing the results in Figure 2 and Figure 3, it can be seen that the positional isomers 3-ADON and 15-ADON could not be separated chromatographically when using Agilent Extend-C18 or Waters UPLC BEH amidel columns. This was consistent with the results by Girolamo et al. [23]. Cortex C18 (2.1 mm × 100 mm, 1.6 μm) and Poroshell HPH-C18 (2.1 mm × 100 mm, 2.7 μm) columns were not able to achieve the full chromatographic separation of 3-ADON and 15-ADON. This also agrees with the study of Jin et al., which showed that the maximum chromatographic resolution of these two compounds was obtained upon using the ZORBAX Eclipse Plus column under optimized gradient conditions [24]. However, stable isotope dilution was used in their study, and the degree of separation between the two peaks was 0.3. To separate isomeric mycotoxins in one chromatographic run, different columns were tested in the study of Rausch et al. [38]. Finally, Raptor FluoroPhenyl (2.1 mm × 50 mm, 2.7 μm) and Raptor Biphenyl (2.1 mm × 50 mm, 2.7 μm,), connected in series and equipped with a FluoroPhenyl (2.1 mm × 50 mm, 2.7 μm) guard column cartridge, were tested for the chromatographic separation of 3-ADON and 15-ADON with a poor peak resolution. These results further support the idea that the good chromatographic separation of 3-ADON and 15-ADON is a challenge. 

Interestingly, From Figure 2c, we can note the YMC-TRIART diol-HILIC column exhibited a tendency to separate 3-ADON and 15-ADON. However, the degree of separation between the two peaks was below 0.5, showing a poor peak resolution. In the initial stage of the infusion experiment, a mobile phase consisting of 5 mM ammonium acetate (A) and methanol (B) was used for each toxin. The elution programme was set as follows: 15% B (initial), 15–90% B (0–12 min), 90% B (12–25 min) and 90–15% B (25–28 min), followed by a 2 min re-equilibration period. Further studies are ongoing to explore the underlying possibility of distinguishing these two isomers. Thus, the mobile-phase gradient also required optimization. In view of this, a 12 min linear gradient programme was applied as follows: 0–1 min, 20% B; 1–2 min, 20–50% B; 2–8 min, 50–100% B; 8–10 min, 100% B; 10–13 min, 100–20% B; and 13–15 min, 20% B. The most striking result to emerge from Figure 2d is that this column gave an optimal chromatographic separation and a delay in the peak time. The chromatographic peaks eluted early, which often leads to significant interference from complex biological samples and affects the accuracy of mass spectrometric analyses. The previous research has confirmed that the separation value would increase when choosing a longer column [39]. Therefore, a longer chromatographic column of the same type was used in this study. As can be seen in Figure 3b, when using the YMC CHIRAa ART Cellulose-sc column (3.0 mm × 250 mm, 3 µm), the retention times of 3-ADON and 15-ADON were 10.06 and 8.80 min, respectively, and the degree of separation between the two peaks was above 1.5, showing a better separation effect than the short column. These are key useful finding indicating 3-ADON and 15-ADON achieved a full chromatographic separation.

### 2.2. Optimization of MS-MS Conditions

In order to select suitable and effective columns, precursor-product ions (397.0 > 307.1 and 397.0 > 173.1 for 3-ADON and 356.1 > 320.9 and 356.1 > 136.9 for 15-ADON) were employed in this study. Furthermore, the precursor ions at *m*/*z* 397.0 and 356.1 observed using the Agilent Extend-C18 (3.0 mm × 150 mm, 3.5 µm) column and the YMC CHIRAa ART Cellulose-sc column (3.0 mm × 250 mm, 3 µm) were defined as method A and method B, respectively. The ratio (the peak area of qualifying ions/the peak area of quantifying ions; Table 1) of 3-ADON and 15-ADON was above 15, showing that the precursor ions at *m*/*z* 397.0 and 356.1 were suitable for the analysis of the two compounds. In addition, we attempted to utilise the deprotonated [M−H]^−^ ion for the separation of 3-ADON and 15-ADON using the YMC CHIRAa ART Cellulose-sc column. Afterward, the optimization of the product ions was conducted at different collision energies to obtain the ideal MS/MS conditions. Fortunately, the ratio of 3-ADON and 15-ADON was 47% (Table 1), indicating that the analytical sensitivity for the two compounds was greatly improved compared with that of method A. In conclusion, the precursor-product ions were 339.1 > 137.1 and 339.1 > 261.1 for 3-ADON and 15-ADON, respectively, which were employed in the accurate determination and quantification of both ADONs. In the present study, this method was defined as method C.

### 2.3. Selectivity

In this study, the selectivity of this approach was chosen to evaluate obvious sources of interference at retention times in blank samples. The different retention times of DON, 3-ADON and 15-ADON were analysed by representative chromatograms of a standard mixture, as shown in Figure 3. It is apparent from Figure 3a that positional isomers 3-ADON and 15-ADON could not be separated chromatographically because of the low separation efficiency of this C18 column for method A. However, Figure 3b,c illustrates exactly that the positional isomers 3-ADON and 15-ADON could be sufficiently chromatographically separated by methods B and C. The retention times in the chromatograms of the blank samples showed high levels of repeatability without interfering peaks, which indicated that there was a good selectivity of these two methods.

### 2.4. Matrix Effect

The experiment was conducted according to the described method as previously reported [19]. The reliability and accuracy of the developed method could be affected by the matrix effect (ME). Generally, the reproducibility of the method would be confirmed, when the SSE is between 80% and 120% [40]. As shown in Table 2, the SSE for DON with methods B and C was 86.5%, indicating that there was no obvious ME. Furthermore, both 3-ADON and 15-ADON suffered no suppression in wheat, and the SSEs were between 81.4% and 95.2%. To minimise the ME and ensure quantitative accuracy, it is necessary to establish matrix-matched calibration curves to calculate the mycotoxin contents in wheat.

### 2.5. Method Validation

An overview of the linearity, retention times and correlation coefficients of DON, 3-ADON and 15-ADON for these two methods is shown in Table 2. The coefficient of determination (R^2^) ranged from 0.9958 (15-ADON) to 0.9992 (DON) for method B and from 0.9966 (15-ADON) to 0.9992 (DON) for method C, which demonstrated good linear relationships for all tested analytes.

On the one hand, the sensitivity of this method was assessed by measuring the LODs and LOQs in this study. LODs and LOQs can be directly measured from chromatograms. By testing, we obtained the following results. The LODs of DON, 3-ADON and 15-ADON were 4 μg/kg, and the LOQs of DON, 3-ADON and 15-ADON were 8 μg/kg for methods B and C. In a previous study (defined as method A in this study), the LOQs of DON, 3-ADON and 15-ADON varied between 5.0 μg/kg for DON and 10.0 μg/kg for 15-ADON. The values of the LODs and the LOQs for 3-ADON and 15-ADON were lower as described by Rausch et al. [24]. In their study, the LODs for 3-ADON and 15-ADON were 12 and 6 μg/kg, respectively, and the LOQs were determined for 3-ADON (40 μg/kg) and 15-ADON (20 μg/kg). In conclusion, based on the comparison of the LODs and the LOQs in our findings with those in the earlier study, the present method is more sensitive.

**Figure 4:**
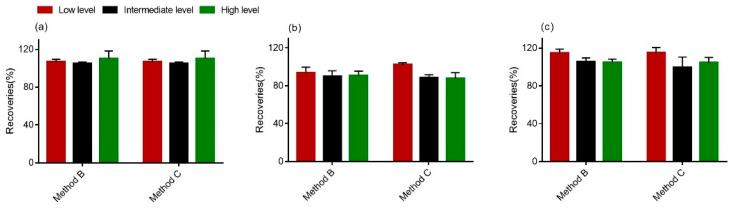
Comparison of the recoveries of DON (**a**), 15-ADON (**b**) and 3-ADON (**c**) in wheat. The high level was 1000 ng; the intermediate level was 500 ng; and the low level was 50 ng.

Furthermore, the intra- and interday precisions (%RSD) were determined. With respect to the intra- and interday precision of the method, standards (50 ng/mL, 500 ng/mL and 1000 ng/mL) were added. Importantly, the RSD_r_ values for all of the samples were less than 15%, whereas the overall RSD_R_ values were found to be higher than the RSD_r_ values, but still less than or approximately equal to 20%, which indicates the stability of this method (<20%) [40]. From the data in Figure 4, we could see that recoveries for all tested analytes ranged from 83.5% to 118.8%, suggesting that the procedure of clear-up met the requirements of multi-mycotoxins determination in wheat [40]. 

### 2.6. Sample Analysis

The established procedures of the clean-up and the LC-MS/MS method were performed in the analysis of DON, 3-ADON and 15-ADON produced by three fungal strains in potato dextrose agar (PDA) and wheat media to be compared with methods A, B and C.

**Figure 5:**
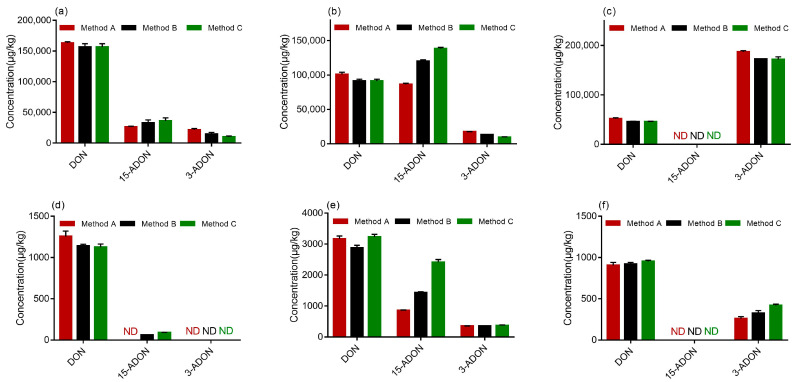
Contamination levels of DON, 3-ADON and 15-ADON determined by three analytical methods: (**a**) strain 5035 cultured in potato dextrose agar (PDA); (**b**) strain PH-1 cultured in PDA; (**c**) strain F-1 cultured in PDA; (**d**) strain 5035 cultured in autoclaved wheat media; (**e**) strain PH-1 cultured in autoclaved wheat media; (**f**) strain F-1 cultured in autoclaved wheat media (μg/kg).

The results are demonstrated in Figure 5, and all the samples were confirmed by the verification of the ion ratios of the quantifier ion to the qualifier ion. According to the test results, three strains of *F. graminearum* produced three kinds of toxins on PDA, namely DON, 3-ADON and 15-ADON. All the strains (PH-1, F-1 and 5035) were capable of producing DON on PDA, which was observed in varying amounts. As shown in Figure 5a–c, there was no significant difference between the DON concentrations determined by methods A, B and C. Similarly, As shown in Figure 5a, strain 5035 produced more DON than 3-ADON or 15-ADON when determined by methods A, B and C. Figure 5b demonstrates that strain PH-1 produced comparable levels of DON and 15-ADON when determined by method A while more 15-ADON than DON was produced by strain PH-1 when determined by methods B and C. Moreover, methods B and C determined the production of less 3-ADON by strain PH-1 on PDA than method A. The results indicated that strains PH-1 and 5035 were 15-ADON producers. Generally, mycotoxin production does not depend on the analytical method used. A possible explanation for this might be that different methods may provide different analytical results based on their performance. Therefore, the result determined by method A could not be accurate, because it could not separate 3-ADON and 15-ADON effectively. Furthermore, the different performances of methods can lead to relevant misinterpretations of chemotypes. It was obvious that method B and Method C developed in this study are more suitable for the chemotype assay of FGC strains than method A. Furthermore, it was observed from Figure 5c that more 3-ADON than DON was produced by the F-1 strain on PDA and 15-ADON was barely detected. Therefore, strain F-1 was of the 3-ADON chemotype.

Again, the chemotype should be determined on PDA; otherwise, the different abilities of strains to grow on wheat and produce mycotoxins could lead to misinterpretation. However, an experiment was carried out to determine whether the developed method could be applied to determine 3-ADON and 15-ADON accurately in wheat samples. Upon comparing the quantities of mycotoxins produced in autoclaved wheat by the three strains, strain 5035 did not produce 3-ADON and 15-ADON at detectable levels when determined by method A, while it produced 15-ADON at low levels when determined by methods B and C, as highlighted in Figure 5d. These results could partly prove that the method developed in the present study is more efficient in the determination of 3-ADON and 15-ADON. We observed from Figure 5e that more 15-ADON than 3-ADON was produced by strain PH-1 on wheat media, which was identical to the results identified on PDA. In addition, Figure 5f presents that strain could not produce 15-ADON in wheat media. Overall, the method developed in this study, including methods B and C, are suitable for the chromatographical discrimination and quantities determination of 3-ADON and 15-ADON from *F. graminearum* in wheat.

## 3. Conclusions

The FGC can be divided into 3-ADON and 15-ADON according to the different types of toxicants produced. Considering that 15-ADON causes the highest toxicity compared to 3-ADON in human intestinal cells, it is necessary to improve the analytical method for the separation of 3-ADON and 15-ADON to conduct comprehensive exposure assessments. Generally, the survey of contamination levels of mycotoxins in agricultural commodities requires the analysis of numerous samples. Therefore, there is a need for a simple and quick approach to reducing the handling time. The present study was designed to determine 3-ADON and 15-ADON in wheat to conduct a comprehensive exposure assessment. In the present study, an LC-MS/MS method coupled with a chiral column for the simultaneous determination of DON, 3- ADON and 15-ADON was investigated. This method was suitable for the determination of DON and DON derivatives in numerous wheat samples. Moreover, the most obvious finding to emerge from this study is that the chromatographic separation of 3-ADON and 15-ADON was achieved. The LODs of DON and DON derivatives were 4 μg/kg, and the LOQs were 8 μg/kg in wheat. The toxigenic ability and toxigenic type of Fusarium are affected by many factors, such as the environment and growth matrix. In previous studies, the toxigenic ability of Fusarium was determined by mass spectrometry, but the methods could not reliably determine the toxigenic type. Our study provides a new way to classify the chemotypes of *F. graminearum* strains and will serve as the basis for comprehensive research on exposure assessments. Several limitations of this pilot study need to be acknowledged. The sample size in this study was not considered large enough, and future studies are needed to explore more *F. graminearum* strains and better define the chemotypes using these candidate methods. Despite its limitations, this study certainly suggested that the rapid detection of these two chemotypes of pathogens has important implications for the successful implementation of disease management strategies. 

## 4. Materials and Methods

### 4.1. Chemicals and Apparatus

The standard of DON, 3-ADON and 15-ADON were purchased from Sigma-Aldrich (St. Louis, MO, USA) and stored at −20 °C prior to use. Acetonitrile, isopropanol, hexane, methanol and ammonium acetate were purchased from Merck (Darmstadt, Germany). In addition, all organic solvents and acids were of analytical or HPLC grade. Milli-Q-quality water (Millipore, Billerica, MA, USA) was used throughout the experiments. The Agilent Extend-C18 (3.0 mm × 150 mm, 3.5 μm) column were obtained from Agilent Technologies Inc (Santa clara, CA, USA). The Waters UPLC BEH amidel (2.1 mm × 100 mm, 1.7 μm) column was purchased from Waters (Milford, MA, USA). The YMC -TRIART diol-HILIC (3.0 mm × 100 mm, 1.9 μm), YMC CHI-RAa ART Cellulose-sc (2.0 mm × 100 mm, 3 μm) and YMC CHIRAa ART Cellulose-sc (3.0 mm × 250 mm, 3 µm) columns were purchased from YMC (Kyoto, Japan).

### 4.2. Preparation of Standard Solutions

Stock standard solutions were prepared in accordance with instructions: the standards were diluted in pure acetonitrile at the following concentrations: 1 μg/mL (DON), 1 μg/mL (3-ADON) and 2 μg/mL (15-ADON). All stock solutions were prepared within 4 weeks and stored at −20 °C before analysis. Then, these solutions were diluted in the blank wheat sample extract to obtain matrix-matched standards. Correspondingly, we used blank wheat samples to develop and validate the proposed method.

### 4.3. Fusarium Strains and Culture Conditions

Three FGC strains PH-1 (*F. graminearum*, chemotype 15-ADON), F-1 *(F. asiaticum,* chemotype 3-ADON) and 5035 *(F. asiaticum*, chemotype 15-ADON), were from Huazhong Agricultural University to determine the chemotype diversity within the FGCs determined by different methods [41]. The strains were routinely maintained on PDA plates at 25 °C.

### 4.4. Mycotoxin Accumulation on PDA

Activated strains were inoculated onto PDA plates and cultured at 25 °C for 5 days. Then, a hole punch was used to collect a fungal disc with a diameter of 5 mm from the colony edge, and the centre of a single point was inoculated onto a new PDA plate (15 mL). Strain inoculation was repeated five times, with an uninoculated PDA plate as the control, and then it was incubated at 25 °C for 7 days. The strains were dried and ground to powder and then prepared for extraction.

### 4.5. Mycotoxins in Wheat Culture

PH-1, F-1 and 5035 were used to artificially inoculate wheat culture. In our study, 20 g of wheat and 10 mL of sterile deionized water were added to 250 mL Erlenmeyer flasks, which were autoclaved (121 °C; 20 min). Then, each flask was inoculated with three fungal discs, with an uninoculated wheat media as the control, and each inoculation was repeated five times. After culturing at 25 °C for 21 days, the wheat grains were dried at 50 °C for 3 days, ground to powder and then subjected to trichothecene mycotoxin extraction.

### 4.6. Pretreatment

One gram of powder was accurately weighed, mixed with 10 mL of an 80:20 (*v*:*v*) acetonitrile/water solution and shaken for 20 min. Two millilitres of the supernatant were placed into a centrifuge tube with 0.3 g of MgSO_4_, after that the mixture was shaken for 5 min, and then centrifuged at 4000 rpm for 5 min at room temperature. Then, the obtained supernatant was added into 1 mL of hexane, followed by shaking for 5 min. A further centrifugation step was carried out at 4000 rpm for 5 min, and the underlying solution was collected for the next step. The obtained solution was evaporated under a gentle stream of nitrogen gas at 45 °C and then redissolved in 1 mL of an acetonitrile/water (20:80, *v*/*v*) mixture. The aqueous phase was transferred into a tube and passed through a 0.22 μm nylon filter (Millipore, 13 mm diameter) for further analysis.

### 4.7. LC-MS/MS Analysis

A DIONEX UltiMate 3000 system (Thermo Scientific, USA) was interfaced with a triple quadrupole mass spectrometer (TSQ VANTAGE; Thermo Scientific, San Jose, CA, USA). An Agilent Extend-C18 chromatographic column (3.0 mm × 150 mm) was used for method A, and a YMC CHIRAa ART Cellulose-sc column (3.0 mm × 250 mm, 3 µm) was used formethods B and C. The sample injection volume was 10 μL, the flow rate was 0.35 mL/min, and the column temperature was 300 °C. The mobile phase consisted of mobile phase A (5 mM ammonium acetate) and mobile phase B (methanol). The elution program was set as an isogradient: 70% B (0–16 min). Mass spectrometric analysis was employed in both positive and negative ionization modes by using multiple reaction monitoring (MRM). The following optimized conditions were set for analysis: vapourizer temperature, 250 °C; ion transfer tube temperature, 350 °C; ESI spray voltage, 3 kV; sheath gas pressure, 30 psi; and collision gas (argon) pressure, 1.5 mTorr. Table 1 details the MS/MS parameters of DON, 3-ADON and 15-ADON for the different methods.

### 4.8. ME

The %ME was calculated by building calibration curves in pure solvent and in the blank matrix [42]. The signal suppression and enhancement can be used to estimate the ME. The ME was calculated according to the following equation:

SSE = A/C × 100,(1)

where A is the slope of the spiked extract and C is the slope of the pure standard. Generally, SSE values ranging from 80% to 120% are considered to be acceptable. 

### 4.9. Method Validation

All developed methods can generally be divided into extraction, sample purification, separation and detection steps. Therefore, the performance characteristics of selectivity, ME, linearity, LODs, LOQs, intra- and interday precisions and extraction recoveries need to be considered. Spiked mycotoxin-free samples were used to validate the method in this study.

#### 4.9.1. Calibration and Linearity

The linearity was evaluated by calibration curves constructed by matrix-matched standard solutions, which the concentration ranges for each toxin were as follow: DON, 3-ADON and 15-ADON (4–2000 ng/mL). The coefficient of determination (R^2^) was calculated by means of the least-square approach.

#### 4.9.2. LODs and LOQs

Alternatively, the LOD can be defined as the lowest concentration of the selected blank samples that could produce chromatographic peaks with a signal-to-noise (S/N) ratio of 3. The LOQ was determined as the concentration with an S/N ratio of 10 [43]. In our study, the LOD and LOQ of each toxin in wheat were determined by serial dilution.

#### 4.9.3. Intraday and Interday Precision

The interday accuracy (RSD_R)_ was evaluated by standards solution on 3 consecutive days, whereas the intraday accuracy (RSD_r_) was determined with triplicate analysis of three concentration levels (50 ng/mL, 500 ng/mL and 1000 ng/mL) on the same day for all 3 analytes. The precision of the total method was calculated from the relative standard deviation (%RSD_R_ and %RSD_r_).

#### 4.9.4. Recovery

In order to investigate the recovery of the candidate method, different concentrations (50 ng, 500 ng and 1000 ng) of DON, 3-ADON and 15-ADON were added to DONs-free wheat sample with six replicates. The recovery was calculated by comparing the peak areas of blank wheat samples, to which toxins were added before and after the extraction process.

## Figures and Tables

**Table 1:** MS/MS parameters of DON, 3-ADON, and 15-ADON in multiple reaction monitoring (MRM) mode with different methods.

Methods	Toxins	Adduct Ion	Precursor Ion (*m*/*z*)	Product Ions (*m*/*z*)	Retention Time (min)	Collision Energy (eV)	Ratio ^2^
Method A	DON	[DON + CH_3_COO]^−^	355.1	295.0 ^1^ 247.0	5.54	17	19.7
	15-ADON	[15ADON + NH_4_]^+^	356.1	320.9 ^1^ 136.9	6.95	12	44.9
	3-ADON	[3ADON + CH_3_COO]^−^	397.0	307.1 ^1^ 173.1	6.97	16	39.2
Method B	DON	[DON + CH_3_COO]^−^	355.1	295.0 ^1^ 247.0	6.38	17	77.5
	15-ADON	[15ADON + NH_4_]^+^	356.1	320.9 ^1^ 136.9	8.81	12	76.4
	3-ADON	[3ADON + CH_3_COO]^−^	397.0	307.1 ^1^ 173.1	10.12	16	40.6
Method C	DON	[DON + CH_3_COO]^−^	355.1	295.0 ^1^ 247.0	6.38	17	77.5
	3-ADON	[DON + H]^+^	339.1	137.1 ^1^ 261.1	8.80	18	47.7
	15-ADON	[DON + H]^+^	339.1	137.1 ^1^ 261.1	10.06	18	47.7

^1^ quantifying ion; ^2^ the peak area of the qualifier in units of the percent of the quantifier.

**Table 2:** Linearities, correlation coefficients, SSEs (%), limits of detection (LODs), limits of quantification (LOQ), (%) RSD_R_ and (%) RSD_r_ of DON, 3-ADON and 15-ADON in wheat.

	Toxins	Linear Range (ng/mL)	Correlation Coefficient (R^2^)	(%) SSE	LOD (ng/mL)	LOQ (ng/mL)	(%) RSD_r_	(%) RSD_R_
Method B	DON	4–2000	0.9992	86.5	4	8	0.28	6.06
	15-ADON	4–2000	0.9958	95.2	4	8	2.19	16.2
	3-ADON	4–2000	0.9985	93.3	4	8	1.85	8.03
Method C	DON	4–2000	0.9992	86.5	4	8	0.28	6.06
	15-ADON	4–2000	0.9966	81.4	4	8	2.55	18.8
	3-ADON	4–2000	0.9991	93.6	4	8	5.19	11.8

## Data Availability

The data presented in this study are available on request from the corresponding author.
